# Virtual and Augmented Reality in Spine Surgery: An Era of Immersive Healthcare

**DOI:** 10.7759/cureus.43964

**Published:** 2023-08-23

**Authors:** Sazid Hasan, Alex Miller, Devan Higginbotham, Ehab S Saleh, Scott McCarty

**Affiliations:** 1 Department of Orthopedic Surgery, Oakland University William Beaumont School of Medicine, Rochester, USA; 2 Department of Orthopedic Surgery, Beaumont Hospital, Royal Oak, USA; 3 Department of Orthopedic Surgery, Wayne State University Detroit Medical Center, Detroit, USA

**Keywords:** residency training, augmented reality surgical navigation, virtual reality (vr), spine surgury, ai & robotics healthcare

## Abstract

In the dynamic realm of spinal surgery, the integration of virtual reality (VR) and augmented reality (AR) technologies is heralding a transformative era. These cutting-edge tools are not only reshaping the training landscape for surgical trainees, offering immersive and interactive experiences but are also enhancing the surgical precision of seasoned professionals in the operating room. While the potential of VR and AR is vast, their adoption is tempered by significant costs and challenges in seamless integration. As the spinal surgery community looks ahead, it becomes imperative to emphasize the validation, reliability, and thorough cost-benefit analysis of these technologies. This article delves into the current applications, benefits, challenges, and future trajectories of VR and AR in spinal surgery, underscoring their pivotal role in the evolution of immersive healthcare.

## Editorial

Introduction

Spinal surgery represents a unique convergence of precision, coordination, and extensive anatomical knowledge. The steep learning curve and the high stakes of error are well-known challenges in this field. [1]. Yet, the continuous forward march of technology has produced tools like virtual reality (VR) and augmented reality (AR) that not only assist in surmounting these challenges but also enable exciting new possibilities in minimally invasive and complex procedures [2]. These technological advancements, often associated with the gaming industry and the realm of science fiction, are becoming integral to the world of spinal surgery, in both training and practice [2].

VR has the potential to create highly accurate, immersive simulations, thereby crafting a realistic training environment for surgical residents outside of the operating room. These technologies offer unique advantages to surgical training, such as the ability to review quantified performance measures using sophisticated analytics and algorithms. Additionally, VR technology immerses the trainees in the surgical process via head-mounted displays and handheld devices with haptic feedback, enhancing the realism of the experience. By rehearsing surgical procedures in the simulation environment, trainees have the opportunity to introduce and develop surgical skills in a flexible setting.

In other orthopedic surgery subspecialties, VR technology's value is already well-established, particularly in arthroscopy training. Compared to traditional training methods, VR provides distinctive advantages like three-dimensional anatomy visualization, simulation of inter-operative complications, and performance recording and analysis. A myriad of studies corroborates the effectiveness of VR in enhancing surgical performance and outcomes.

Current developments

VR and AR in Spinal Surgery: From Simulation to Operation

Though similar to VR and AR, navigation technologies have been effectively used in spinal procedures, notably pedicle screw placements. Current intraoperative navigation platforms (such as Medtronic StealthStation and Mazor) utilize monitors to provide visual feedback coupled with CT data. AR goes a step further by superimposing a layer of digital information onto the real world, essentially blending the virtual and the physical. There are several emerging products that have begun the transition into AR (such as the Xvision system by Augmedics), integrating headset-mounted visual feedback. This allows for a visual overlay directly onto the patient. AR holds the potential to employ holographic imaging to guide surgeons, though no commercially available products are currently available.

With improved visual cues offered by VR and AR, the surgeon gains provide an additional layer of information when encountering difficult anatomy that could make the difference between a successful surgery and a problematic one [3-5]. Despite this, many surgeons are reluctant to utilize technological assistance for familiar procedures. Yet, early research supports the utility of this new technology. In a study examining the effectiveness of AR in thoracic pedicle screw placement, the simulator demonstrated a performance accuracy that was on par with traditional methods [6]. These findings underscore the effectiveness of the early iterations of these technologies in improving surgical accuracy.

With VR, surgeons are afforded an immersive, interactive environment that supplements the baseline surgical visualization. One of the key advantages of VR is that it can provide visual cues and feedback to the surgeon in real-time during the procedure, increasing surgical precision [4]. Studies have shown that VR simulations can reduce errors in pedicle screw placement by up to 53.7%, underscoring its potential in enhancing surgical accuracy [4].

Beyond Pedicle Screws: The Broadening Scope of VR and AR in Spinal Surgery

The applications of VR and AR technologies in spinal surgery extend beyond pedicle screw placements. These cutting-edge tools are finding use in a variety of spinal procedures, from decompression to tumor resection to deformity correction surgeries. AR can provide surgeons with real-time, 3D visualizations of the patient's spinal anatomy during spinal decompression surgeries, allowing for more accurate identification and resection of compressive pathology. Similarly, in tumor resection procedures, VR and AR can help visualize the tumor's exact location and its relationship with surrounding structures, enabling a more targeted and safer surgical approach. As more patients request less-invasive and minimally invasive treatment options, AR and VR-assisted approaches in the field of endoscopic spine surgery may help reduce the learning curve to this newly emerging procedure.

In spinal deformity correction surgeries, these technologies can aid in preoperative planning by providing 3D visualizations of the patient's spinal alignment and providing more detailed and reliable osteotomy landmarks. The AR overlay can help surgeons visualize the final alignment before making any surgical cuts, leading to more accurate and reproducible surgical execution. Studies have shown that the use of VR and AR in these procedures can lead to better patient outcomes, with fewer complications and revisions [2-5].

Integration of VR and AR into surgical practice: opportunities and challenges

The integration of VR and AR technologies into everyday surgical practice presents unique opportunities and challenges. On the one hand, these technologies have shown significant potential in improving surgical outcomes by increasing precision, reducing complications, and enhancing patient safety [2]. On the other hand, their adoption in surgical practice has been limited by several factors. 

One of the primary obstacles to wider adoption is the high cost associated with these technologies. The adoption of VR and AR technologies often requires a substantial initial investment, primarily due to the cost of hardware, software, and maintenance [2,7]. This can be seen with programs such as Augmedics Xvision which plans to sell the product to hospitals at about $200,000 to $300,000 [8]. The system costs itself costs about $179,000 but additional training, onboarding, and disposable items per-procedure costs must be accounted for. Furthermore, these systems need to be integrated into the existing surgical workflow, which can be a complex process requiring additional training to both staff and surgeons and initial decreases in efficiency while accommodating changes to established surgical routines. However, when considering the long-term value, these technologies can lead to reduced operating times, decreased surgical trauma, and fewer revision surgeries, which can offset the initial costs over time [2]. Watkins et al. (n=100) reported that the approximate cost of the navigation systems used in their studies totaled about $475,000 in 2010 with the Vector Vision-BrainLA around $225,000 and $250,000 for Arcadis Orbic-Siemens [9]. However, the study concluded that the technology saved the institution about $71,286 per 100 cases in costs which would have been incurred in longer operating times and revision procedures [9].

The integration of VR into residency training programs has seen a mix of enthusiasm and hesitation [2]. In a survey of orthopedic residency programs, only 9.8% used VR for arthroscopic training [2]. The high initial costs associated with high-fidelity simulators have resulted in variability in simulator availability across institutions. Currently, no standardized curricula for VR/AR surgical tools are endorsed by major orthopedic specialty organizations. The success of the Fundamentals of Arthroscopic Techniques (FAST®) bench top trainer serves as an example of a coordinated effort between the American Academy of Orthopaedic Surgeons (AAOS), Arthroscopy Association of North America (ANA) and American Board of Orthopaedic Surgery (ABOS) in developing a surgical simulation product that has been widely adopted by residency and fellowship programs. As access to AR/VR expands, validated assessments of trainee skills will be important for assessing the utility of these simulators and justifying the capital investment in these often-expensive technologies. When considering potential improvements in patient safety, increased efficiency of intraoperative teaching, and improved faculty confidence in trainee involvement, these benefits must be quantified when considering the purchase and maintenance of these simulators.

The global disparity in access to advanced medical technologies is a well-documented challenge [10,11]. While developed nations are rapidly integrating AR/VR into their medical practices, low socio-economic countries face unique hurdles in adopting these innovations. The primary barrier is the high initial investment required for VR and AR systems, which often becomes prohibitive for healthcare institutions operating on limited budgets [10]. However, the potential benefits of VR and AR in spinal surgery training and practice make a compelling case for exploring cost-effective solutions. Collaborative initiatives between developed and developing nations, non-profit organizations, and technology providers can pave the way for subsidized systems or shared resources [11]. For instance, mobile-based VR solutions, which are relatively more affordable, can be an entry point for institutions in resource-limited settings [10]. Furthermore, international training programs can leverage VR and AR to provide remote training, reducing the need for physical travel and associated costs.

Conclusion: future developments and directions

Despite these challenges, as these technologies continue to evolve and become more streamlined, it's expected that their use in spinal surgery will become more widespread. With improved accessibility and reduced costs, VR and AR technologies have the potential to become integral and indispensable tools in spinal surgical practice, helping surgeons navigate the complex anatomical landscape of the spine with increased confidence and precision. As we venture further into the 21st century, the influence of VR and AR in spinal surgery is set to grow. We expect that as more data becomes available that supports the benefits of VR and AR in enhancing surgical outcomes, their use in spinal surgery is expected to increase. Careful assessment and consideration are needed to ensure the potential of this technology is harnessed without compromising the rigorous standards of surgical practice. As technology is refined and the implication of its utilization is better understood, it becomes clear that the future of spinal surgery may well be alongside the virtual.
